# Negative childhood events and the development of the anorexic voice: A grounded theory

**DOI:** 10.1111/papt.12416

**Published:** 2022-07-27

**Authors:** Jet Morrison, Marc O. Williams, John R. E. Fox

**Affiliations:** ^1^ School of Psychology Cardiff University Cardiff UK

**Keywords:** adverse childhood experiences, anorexia nervosa, anorexic voice, eating disorders, trauma

## Abstract

**Background:**

Many individuals diagnosed with anorexia nervosa (AN) describe their disorder as being represented by an internal ‘anorexic voice’ (AV). Previous studies have identified associations between eating psychopathology and multifarious forms of adverse life experiences.

**Aims:**

This study explores the relationship between adverse experiences in childhood and the development of the AV.

**Materials and Methods:**

Twelve women who had the experience of the AV in the context of a diagnosis of AN took part in semi‐structured interviews. The interview data were analysed using a constructivist grounded theory methodology.

**Results:**

Participants recalled feeling unsafe in a variety of relationships and a theory was constructed in which the AV provides a sense of conditional safety, becoming increasingly hostile and belittling when it is disobeyed, revealing similar characteristics to abusers and bullies in childhood.

**Discussion:**

Findings are related to the broader literature on the link between trauma and eating disorders, and to existing theories of internal voices.

**Conclusions:**

The findings have implications for a trauma‐focused approach when working with the AV.

**Practitioner Points**

- This study explored the anorexic voice and its relation to early life adversity in women who have been diagnosed with anorexia nervosa
- The anorexic voice appears to share characteristics of abuse and bullying experiences in childhood
- Practitioners should consider using therapeutic approaches that address relational trauma with those who experience an anorexic voice in the context of anorexia nervosa

## BACKGROUND

Diagnostic manuals characterize anorexia nervosa (AN) by restriction of calorific intake in the context of fear of weight gain/body change and/or behaviour interfering with weight gain (Diagnostic and Statistical Manual of Mental Disorders [DSM‐5]; American Psychiatric Association, 2013). Recommended interventions for AN consist of weight restoration and eating disorder (ED) focused psychotherapies (National Institute for Health and Care Excellence, 2017). Many individuals do not improve after treatment, and even after initial successful outcomes the relapse rate is high (Dobrescu et al., 2019; Zipfel et al., 2014). This could suggest a lack of understanding of the underlying mechanisms in AN, such as the anorexic voice (AV; Pugh, 2016).

The AV is characterized as a hostile internal dialogue related to eating, shape, and weight (Pugh, 2016). It is thought to be a key psychological component that contributes to the development and maintenance of AN (Higbed & Fox, 2010; Pugh, 2016; Tierney & Fox, 2010). Similar voices have been reported in other ED groups, including bulimia nervosa (Noordenbos et al., 2014; Pugh et al., 2018), suggesting this phenomenon may be a transdiagnostic feature across eating psychopathology. Conceptual tensions surround the AV, with some describing it as a metaphorical experience or internal thoughts (Graham et al., 2019) and others suggesting it represents a separate entity related to the self (Pugh & Waller, 2017). Preliminary work suggests that an intervention targeting the AV may be acceptable to patients with eating disorders and could increase motivation for change, hope for recovery, and decrease the extent to which patients identify with the AV (Hibbs et al., 2020).

In the early stages of AN, the AV is perceived as comforting and safe however becomes critical and dominant, imposing high expectations on individuals, and demanding obedience from them (Tierney & Fox, 2010, 2011; Williams & Reid, 2010, 2012). Living with AV is experienced as a ‘battle’ (Williams et al., 2016) and is associated with negative feelings such as loneliness (Tierney & Fox, 2010, 2011; Williams & Reid, 2010, 2012). Despite this, individuals appear unwilling to leave the relationship with the AV (Tierney & Fox, 2010, 2011; Williams & Reid, 2010, 2012). It is unclear why the AV changes from being comforting to increasingly toxic (Tierney & Fox, 2011).

The AV may stem from early experiences of relational criticism and/or punitiveness which are internalized (Fox et al., 2012). A variety of childhood trauma types have been studied in relation to eating disorders. Molendijk et al. (2017) conducted a meta‐analysis exploring three types of childhood maltreatment: physical, sexual, and emotional abuse, concluding that all three were associated with the presence of AN, bulimia nervosa, and binge eating disorder. As for AN specifically, Molendijk et al. reported stronger evidence for the association between childhood maltreatment and the binge‐purge subtype than the restricting subtype. Other types of adverse childhood experiences appear to be related to the development of AN, such as being bullied (Copeland et al., 2015).

Pugh and colleagues (2018) suggest internal voices arise from detachment from internal events related to early trauma, citing the trauma‐dissociation model (e.g., Longden et al., 2012). Longden et al. argued that voice‐hearing across different clinical presentations is best understood as dissociation from aspects of the self, or relationships between the self and others, as a result of adverse experiences, citing empirical support such as associations between measures of voice‐hearing, trauma, and dissociation. Pugh et al. reported findings from a mixed ED group that the perceived power of the AV is positively related to childhood emotional abuse, but no other early traumas, and this association was partially mediated by dissociation. This is consistent with work suggesting that emotional abuse is most strongly associated with emotional regulation disruption and AN symptoms amongst people with AN (Racine & Wildes, 2015). However, Pugh et al. included a mix of ED diagnoses; thus, it is unclear to what extent their findings apply to AN specifically. Furthermore, methodological restrictions, such as the use of self‐report measures, may have limited the range of childhood adversities recalled by individuals as well as other trauma‐related factors which may act as mediators in the relationship between childhood trauma and AV.

Experiencing childhood adversity has been suggested to increase sensitivity to interpersonal stress due to fears of rejection and abandonment, and difficulty establishing trust with others (Messman‐Moore & Coates, 2007). Kong and Bernstein (2009) reported that 90% of their sample of individuals with ED had experienced at least one kind of abuse or neglect in childhood. Despite this, the research is still inconclusive; some studies have found associations whereas some have not, and some researchers suggest associations are overstated (Polivy & Herman, 2002; Smyth et al., 2008; Villarroel et al., 2012). To better understand the possible relationship between childhood adverse experiences and AN, some authors have considered possible mediators such as dissociation (Pugh et al., 2018), anxiety and depression (Rabito‐Alcón et al., 2021) insecure attachment (Illing et al., 2010), and emotional dysregulation (Mills et al., 2015). For example, Rabito‐Alcón et al., 2021 suggested that eating disorders may have a variety of functions; eating disorders may function to suppress overwhelming emotions resulting from prior trauma, or may serve as a way of regulating other emotional experiences stemming from trauma, such as anxiety and depression. Furthermore, Tasca and colleagues (2009) found that the association between attachment anxiety and AN was mediated by emotional reactivity, and the association between attachment avoidance and depressive symptoms in patients with AN was mediated by dissociation. It appears that childhood adversity could predispose individuals to develop AN for a variety of reasons.

The current research aims to qualitatively investigate whether individuals perceive there to be an interaction between the AV, emotions, and childhood experiences, and to generate new theories to explain any associations. Understanding why and how AV, emotions, and childhood interact may further our understanding of the often‐intractable nature of AN and low rates of recovery (Pugh et al., 2018). An inductive qualitative methodology is well suited to explore this research aim and qualitative approaches have been welcomed in AN research (Williams et al., 2016). Grounded theory is used to provide a framework to identify categories of data and to integrate them into theory describing emergent processes (Willig, 2013).

## METHODS

### Design

Semi‐structured interviews were used to collect data which was analysed using principles of grounded theory (Glaser & Strauss, 1967). This study employed a constructivist grounded theory methodology (Charmaz, 2006), which allows theories to emerge from data (Charmaz, 2006). Using a process of ‘constant comparison’, theory generation becomes progressively more abstract at each level of comparison, for example, data were compared with data, data with categories, and categories with categories (Charmaz, 2006). Data analysis and collection took place simultaneously which allowed for deeper exploration of similarities, differences, and associations between categories within the data (Charmaz, 2006). The constructivist grounded theory highlights the importance of the researcher's role in constructing meanings (Charmaz, 2006). This position argues for flexibility in the analytical process to improve credibility (Creswell et al., 2007) thus reflexivity and transparency are required throughout the research process.

### Participants

Participants were recruited from adverts on social media – Facebook, Twitter, and Instagram. A third‐sector charity advertised the research on their website. Interested individuals were directed to an online survey which was used to outline information about the research (using Qualtrics XM Software, 2019; Qualtrics) and assess inclusion suitability (see Table 1). To minimize the chances of recruiting participants currently experiencing very high physical risk from their eating disorder, participants with a body mass index (BMI) under 14 were not included. Individuals with psychosis were also excluded to allow the researchers to focus exclusively on experiences of voices relating to the eating disorder. Potential, suitable, and consenting participants were contacted by the researcher to invite them to an interview. A convenience sampling method was employed.

**TABLE 1:** Inclusion and exclusion criteria for participants

Inclusion criteria	Exclusion criteria
Aged 18 or over Reporting having a diagnosis of anorexia nervosa Currently living in the United Kingdom English‐speaking Consenting to provide contact details	Comorbid diagnosis of psychosis BMI 14 or under Currently admitted as an inpatient

Twelve participants were interviewed. Participants said they had previously received a diagnosis of AN. Some were still receiving support from clinical services and none identified as being ‘recovered’. All participants self‐reported their diagnosis and current weight. Participants were residents in the United Kingdom and all identified as female. Participants' age ranged from 19 to 39 years (mean = 25.3 years). A range of BMIs was included (mean = 17.1). All participants had undertaken higher education (college *n* = 4; undergraduate *n* = 5; and postgraduate *n* = 3). Two women had additional diagnoses, which we have chosen to omit here to ensure anonymity. The sample characteristics are shown in Table 2.

**TABLE 2:** Overview of participants

Participant (pseudonym)	Age	BMI	Ethnicity	Education level	EDE‐Q global score	CTES
Alice	27	20	White other	Postgraduate University	2.8	Parental divorce/separation Parent with depression Major upheaval
Lara	26	21	White British	Postgraduate University	2.4	Family mmber Death Sexual abuse Physical abuse Physical illness
Marie	22	18	White British	Undergraduate University	4.4	Family member death Parental divorce/separation Sexual abuse Major upheaval
Rose	29	16.7	White British	Undergraduate University	2.0	Family member death Parental divorce/separation Sexual abuse Physical abuse Physical illness
Sarah	19	22	White British	College	2.2	Family member death Sexual abuse Physical illness major upheaval
Mandy	39	18	White British	Undergraduate University	0.10	Family member death Parent divorce/separation Major upheaval
Ellie	25	21	White Irish	Undergraduate University	1.5	Parental divorce/separation Sexual abuse Physical illness Major upheaval
Lydia	32	15	Asian/Asian British, Indian	Postgraduate University	4.9	Family member death Physical illness Major upheaval
Hayley	21	15	White British	College	1.6	Sexual abuse Physical illness Major upheaval
Meredith	21	16.5	White British	Undergraduate University	4.0	Family member death Sexual abuse Major upheaval
Lauren	21	20	Black British	College	1.6	Family member death Sexual abuse Physical abuse Physical illness Major upheaval
Emma	22	18	White British	College	2.9	Family member death Sexual abuse Physical abuse Major upheaval

### Measures

Prior to the interview, participants completed the Eating Disorder Examination Questionnaire (EDE‐Q; Fairburn & Beglin, 2008). This measure asks participants about the frequency and severity of eating disorder behaviours and concerns over the previous 4 weeks. A global score can be calculated, and there are also four subscales: restraint, and eating/shape/weight concern. The EDE‐Q has demonstrated good psychometric properties (Luce & Crowther, 1999; Mond et al., 2004). It has been extensively used with a range of eating disorder groups and is considered the gold standard instrument for measuring eating disorder symptomatology in (Berg et al., 2012).

The Childhood Traumatic Events Scale (CTES; Pennebaker & Susman, 1988) assesses childhood traumatic events that occurred prior to the age of 17. Domains include the death of a family member or a very close friend, parental divorce or separation, traumatic sexual experience, direct experience of violence, experiences of extreme illness or injury, and other major upheavals. For each question, the participant is asked to recall the age of trauma, the perceived intensity of the trauma, and whether or not the trauma was confided to others. It has been shown to have good reliability and validity (Pennebaker & Susman), and is sensitive to a diverse range of clinical symptoms following early life trauma (Pennebaker & Susman, 1988; Scheller‐Gilkey et al., 2004; Thakkar & McCanne, 2000).

### Data collection

Data were collected through semi‐structured interviews which provide flexibility and allow for follow‐up prompts. The first author interviewed all participants through Zoom video call. Prior to the interview, participants were directed to the Qualtrics survey where they read the information sheet and indicated their informed consent via a tick‐box. The EDE‐Q (Fairburn & Beglin, 2008) and demographic information was also collected via Qualtrics. The researcher invited participants to interview via email. These data were gathered to situate the sample and provide context about their ED pathology and mood, which may influence their experiences. Three participants had a score higher than four, indicating that most of this sample had relatively low eating pathology at the time of the interview. The CTES (Pennebaker & Susman, 1988) was completed with the researcher after the interview. This questionnaire recorded data on participants' childhood trauma.

Interviews were commenced and lasted between 60 and 90 min. Participants then completed the CTES (Pennebaker & Susman, 1988) with the researcher. The purpose of including the CTES was to describe the kinds of trauma reported by participants (see Table 2). It was administered at the end of the interviews to avoid biasing the information that might be reported by participants. A participant debrief took place after the interview to allow reflection on the research process and to assess risk. Participants were emailed a debrief sheet containing information about additional support services if required.

A semi‐structured interview schedule was developed in line with Charmaz (2014). The collaborative nature of creating the interview schedule provided the opportunity to be reflexive about the nature of the questions. Using grounded theory principles, the interview schedule was adapted three times as ideas and concepts emerged during the early data analysis. This allowed for constant comparisons with subsequent data to identify key processes and other analytic ideas emerging out of the data. To achieve this, three interview schedules were developed by the research team and adapted in line with the participants' experiences. The final interview schedule comprised 15 open‐ended questions with prompts exploring experiences of the AV, negative childhood events, emotions and interactions between the three (Table 3).

**TABLE 3:** Interview Questions

Lots of people with a diagnosis of anorexia nervosa talk about experiencing an internal voice related to anorexia – Is this something you have experienced? Can you tell me more about your experience with the anorexic voice has been? What is this like for you? What does it say to you? Does it remind you of anyone? Does it comment on any aspects of your life? Can you tell me about what life was like for you when you were growing up? Who was at home? Can you describe your relationship with…? Can you give me an example of…? How has that impacted you as a person? How has this impacted your experience of the voice? Could you please tell me a bit more about what school was like for you? What did you like/dislike? How long were you at school? Who do you remember from school? How did you get on with peers at school? Could you please tell me about any negative events you might have experienced? Could you describe if the anorexic voice impacted your childhood in any way? If so, how? Could you tell me more about what life was like for you at the time? Could you please tell me about how people in your family experience emotions? What do you think about that? How has that impacted you as a person? How, if at all, has this impacted your experiences of emotions? Would you be able to tell me how you experience emotions? How do you know you are feeling X? Who would you turn to when you were upset? How would you let others know how you were feeling? What do you do when you are feeling X? How does this fit with your eating disorder? Would you be able to tell me about any important people in the journey of your anorexia? Could you tell me a bit more about how this was experienced as positive/negative? In your opinion, what events/influences might have precipitated the onset of your disorder? Would you be able to describe to me what is helpful/unhelpful about the anorexic voice? Is there something that you might not have thought about before that occurred to you during this interview? Is there something else you think I should know that maybe I have not asked about? Do you have any questions for me?

### Ethical considerations

Full ethical approval was obtained from [redacted] ethics committee before recruitment began.

### Data analysis and coding

Interviews were recorded during the video and transcribed verbatim. Interview data were analysed using Nvivo Software (QSR International, Version 12) which stored raw data and codes. Reflective journals and memos were kept in separate documents. Analysis was a dynamic process where collection and coding occurred in parallel. Data were constantly compared with data and codes as they emerged. For the credibility of the data analysis, the first author consulted the research team to discuss the meaning that was drawn from participants' statements. Every level of coding involved all three authors.

Each transcript was initially coded line by line, remaining open to analytical possibilities and capturing the meaning. As recommended by Charmaz (2006), questions were asked of the data to guide the coding process: ‘What process(es) is at issue here? How can I define it?’ and ‘When, why and how does the process change?’. The constant comparison method was used, in which the first author repeatedly compared codes with each other, and with the interview transcripts, to aid in the clustering of codes into larger and more abstract categories, which then became focused codes. Focused codes help to condense, synthesize, and explain larger segments of data thus indicating the theoretical direction of the data. Focused codes were reviewed, and similar codes collapsed together if deemed appropriate through constant comparison to initial codes and transcripts. Focused codes with the most theoretical weight were lifted into conceptual categories and other focused codes collapsed into these. Theoretical coding was used to identify links between the conceptual categories, codes, and concepts, leading to the development of an interpretive theory and framework. Examples are given in Table 4.

**TABLE 4:** Example of coding procedure

Raw Interview extract *(Sarah)*	Initial coding	Focus coding	Category	Theoretical concept
‘Um, so, I felt very alone and it sort of felt like it was the one thing that I could rely on that would be there for me, um, and that was just sort of talking to me. I guess at that point I had somewhat externalised it because it didn't feel like I was just, you know, talking to myself. Um, but it certainly had like quite a positive characteristic because I felt so alone, and that it was sort of the only thing that was really there for me.’	Feeling alone Relying on voice Voice being there for me Talking with voice Starting to externalizing voice Not feel like talking to myself Describing voice as having positive characteristics Feeling alone; describing voice being supportive	Experiencing loneliness Voice providing similarities to friendship Seeing voice as part of self Positive characteristics of voice Experiencing loneliness Supportive voice	Loneliness Comforting Sharing self with Voice: ‘Evil twin’ Comforting Loneliness Comforting	Internal unsafety ‘You are safe with me’ Self: ‘Evil twin’ ‘You're safe with me’ Internal unsafety ‘You're safe with me’

### Reflexivity

Constructivist grounded theory recognizes the researcher is not a neutral, value‐free observer in the research process (Charmaz, 2014). The researcher brings their own epistemology, perspectives, and interactions from personal and professional experiences. The first author, at the time of undertaking the study, was a 30‐year‐old white Scottish woman undertaking doctoral training in Clinical Psychology, including theoretical teaching in ED and experience working in trauma services. The researcher acknowledges preconceptions and perspectives regarding the impact of trauma in childhood and theoretical understanding of AN. The research was supervised by two white British Clinical Psychologists with experience working clinically with people who have ED. Both supervisors have also conducted their own research using this methodology.

These factors may have compelled the researcher and supervisors' desire to identify emergent theory based on preconceptions and previous knowledge. Using memos and reflective journals pre‐conceived ideas were bracketed off and these influences were mitigated and managed. Regular supervisor research meetings allowed the preconceptions and previous knowledge of all researchers to be bracketed, reducing the potential influence over, for example, the interview schedule development, recruitment, and data analysis.

### Respondent validation

Participants were consulted by the researcher to verify that the results and diagrammatic summary were an accurate reflection of their experiences. A copy of the model was emailed to participants. Responses were incorporated into the final theory. Participants expressed that the findings considerately explained their experiences with their AV, childhood, and emotions. Feedback identified that the results and model reflected their personal experience and were comprehensible.

## RESULTS

**FIGURE 1:**
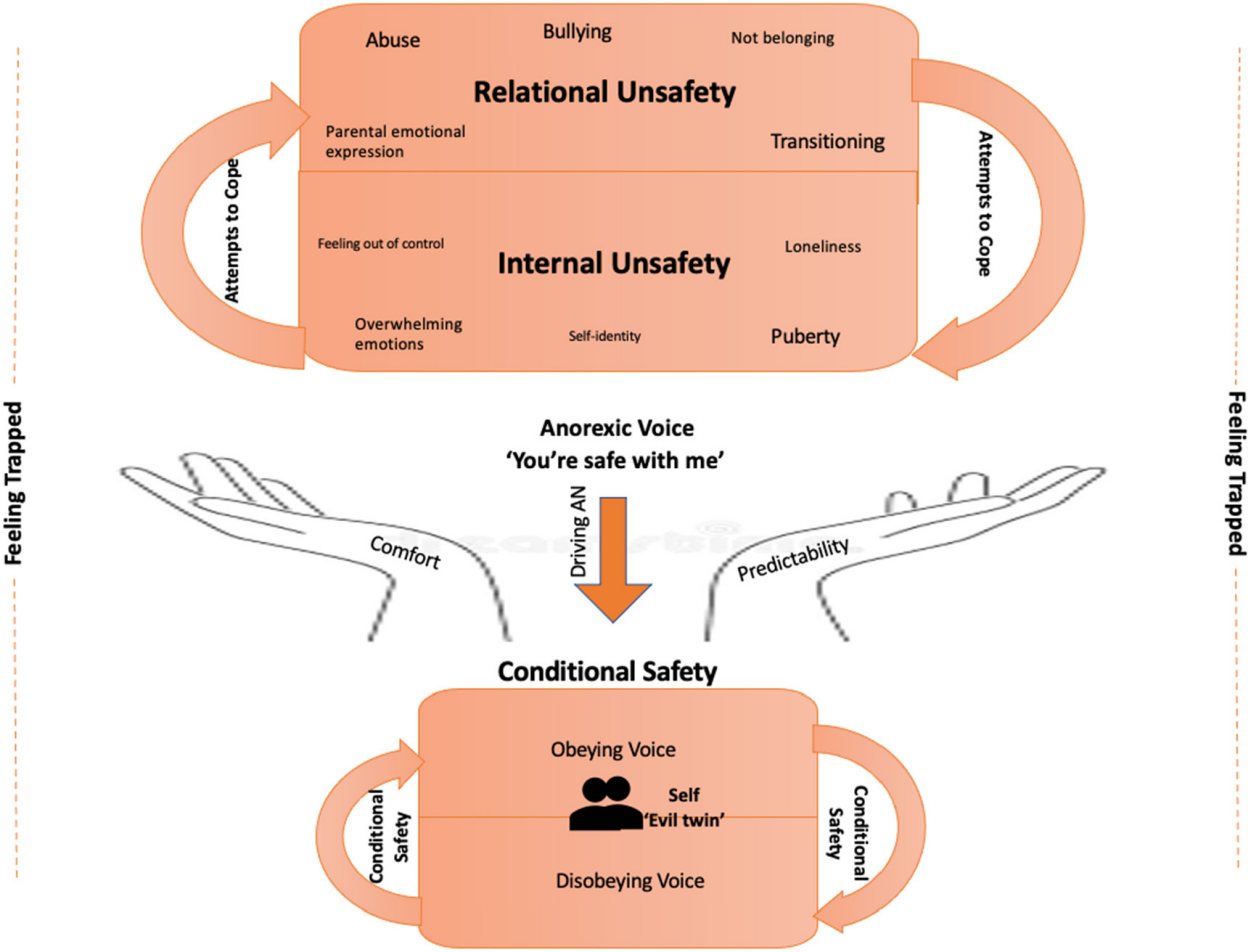
Diagrammatic summary of grounded theory representation of perceived associations with the AV

An explanatory framework of the relationship between childhood experiences, emotions, and the AV was developed through a grounded theory approach (Charmaz, 2006). Each category and concept are described below with illustrative quotes. Figure 1 provides a diagrammatic representation of the theory and the relationship between categories.

### Overview of the model

All participants described experiencing an AV that was predominantly focused on food intake, weight and shape, and exercise regime. Participants recalled feeling unsafe across a variety of relationship‐based experiences in childhood, both at home and school; the researcher termed this feeling ‘relational unsafety’. Examples of relational unsafety were abuse, bullying, and having emotionally erratic parents, and the experiences of meeting new people in an unfamiliar environment in the transition to secondary school. Experiencing interpersonal relationships as unsafe appeared to influence participants' sense of threat from overwhelming internal experiences, including overwhelming emotions, feeling out of control, and loneliness; the researcher termed this ‘internal unsafety’. Puberty was another factor that participants described as generating internal unsafety. Participants described attempting to cope with both types of unsafety with two inter‐related methods: not confiding in anyone and striving for perfection. The AV appeared to provide participants an experience of safety, ‘you're safe with me’; however, this safety was conditional on participants obeying the rules set by the AV. By promising safety, comfort, and predictability the AV was central in driving AN behaviours. When participants did not obey the AV (due to exhaustion, engaging with mental health services, or loved ones), the AV was described as becoming increasingly abusive. This often led participants to succumb to the AV and re‐engage with AN behaviours thus placating the AV. Participants described experiences of feeling trapped in childhood as being mirrored by the experience of feeling trapped by the AV. These key concepts, and how they inter‐relate, will now be described in more detail.

### Relational unsafety

Physical and emotional abuse in childhood were commonly reported by the participants. Those who did not experience physical or emotional abuse described a home life marked by distressing changes to valued support systems, such as those caused by domestic violence, parental illness, divorce, or death in the family. Sexual abuse was noted by many participants on the CTES, with two exceptions, and some participants briefly described sexual abuse in the interviews. None of the participants chose to discuss this in any detail in the interview process.

I would like lean over the bath to like wash my hair if that makes sense she [mum] would like accidentally hit my face on the side of the bath until my nose would bleed. (Lara)

These experiences appeared to generate feelings of uncertainty in participants, or ‘walking on eggshells’ (Rose), and doubt in their ability to cope with situations. Domestic violence and abuse evoked feelings of shame and anger in participants. Participants did not communicate these feelings with anyone for fear of the perceived consequences of asserting themselves. For the participants who experienced abuse, uncertainty overlapped with feelings of dread and fear in the presence of the abuser.

You hear the person coming up the stairs and that fear, that dread. (Emma)

There was a trend in which some participants mentioned a lived experience of one parent being emotionally unpredictable and the other parent being emotionally suppressive. Participants' accounts suggested that the unpredictable parent tended to be abusive; the parent would ‘really randomly really suddenly get like really cross at us for something quite minor’ (Alice). Participants appeared to blame themselves for causing the outburst of emotion. Participants' self‐blame and fear of further provocation of emotional outbursts appeared to inhibit their ability to assert themselves or express their emotions towards their parents. By avoiding communicating their emotions, participants seemed to create a distance between themselves and their parents which felt safer than engaging relationally. However, this led to feeling lonely.

All participants experienced appearance‐focused and emotional/psychological bullying at school. Individuals described attempting to change themselves to placate the bullies. Bullying appeared to confirm self‐beliefs about being a bad person which destroyed self‐confidence. This self‐belief could potentially be related to experiencing relational unsafety at home and developing a tendency to blame themselves for others' behaviour towards them. Despite participants' efforts to change aspects of themselves, the bullying continued. This created a sense of powerlessness, feeling trapped, and a lack of control in the interactions.

Transitioning to high school was seen as a particularly difficult time, evoking overwhelming feelings of anxiety resulting from a loss of familiarity, and predictability from primary school and the sense that they did not belong in this new social environment. Not belonging appeared to impact the belief that the participants were different from others in some way, and exacerbated anxiety about interacting with new people in new situations. Participants retreated increasingly into themselves during this period and appeared to disengage with the world around them. Feeling relationally unsafe was intensified by puberty, which enhanced the perception of being different from others. Participants spoke about feeling uncomfortable in their changing body, feeling unprepared for puberty, and interpreting their changing body as becoming fat.

### Internal unsafety

Participants described how experiencing interpersonal relationships as unsafe appeared to influence their internal experience. Overwhelming feelings were described as co‐occurrence of a variety of emotions, including anxiety, anger, fear, shame, guilt, self‐blame, and disgust. Participants reported experiencing emotions deeply and finding these emotions confusing and intensely unpleasant. This highlights a potential parallel between participants experiencing one parent as unpredictable and untrustworthy due to their emotional expression, which might have fostered an untrusting relationship with their own emotions.

I've always had an issue with like I feel things very deeply and I I I used to hate that I used to think that that was like a bad thing and I used to get overwhelmed by it quite easily (Rose)

Participants experienced loneliness in two ways. First, loneliness was something imposed on them by others, for example, as a consequence of emotional/psychological bullying. Participants also described loneliness as something self‐imposed, resulting from their withdrawal from the social world. One participant described experiencing poor emotional connection and hostile relationships with others in childhood as creating a ‘void of emptiness’ (*Lydia*) within her. The ‘void of emptiness’ appeared to lead participants to seek comfort in the AV:

…you would then revert to your inner self go away and lock yourself up and spend time and that loneliness becomes bigger and then to fill that empty void you then start to create an inner friend in a voice which then comes becomes part of you or part of the world. (Lydia)

### Conditional safety: ‘You are safe with me’

Although the AV was described as providing an antidote to relational and internal unsafety, participants described this safety as conditional. The AV had a ‘behavioural code’ (Sarah) focused on calorie intake, body shape, and exercise, which participants needed to obey, or it would become like ‘a drill sergeant in your head’ (Lara) giving forceful orders. When obeying the AV, participants described it as ‘a guiding light’ (Rose) promising to make them a ‘better person’ (Hayley) which was ‘comforting and reassuring’ (Mandy). In contrast, when disobeyed, participants recalled the AV as becoming increasingly hostile and powerful by mimicking the tone, volume, and comments on appearance or self‐worth received by abusers or bullies.

And it was the same with the eating disorder voice, you know what's going to come next if you don't do what it says. It's almost like that bully, I guess, like that angry parent, angry bully, you are waiting for it […] it goes from zero to one hundred in terms of if you don't do what it's saying. (Emma)

Participants described a relationship with the AV that mirrored the descriptions of dynamics that some had experienced when groomed in childhood. For example, the AV was perceived as using strategies to keep the individual dependent: issuing threats and bribes, isolating participants from others and exploiting vulnerabilities, for example, low self‐confidence. The AV was also described as encouraging the individual to keep it a secret from others. As previously mentioned, all participants experienced sexual abuse, except for two, and it is therefore possible that the perceived relational dynamics with the AV were influenced by traumatic relational experiences.

It would just be like, it's okay. No one else has to know about it. It's absolutely fine. And, you know, we can do it one time. If you don't like it, we don't have to do it again. It's, you know, it's absolutely fine. It's going to – but you need to do it. (Sarah)

### Self – evil twin

Over time the AV appeared to shape the participants' sense of self‐identity. Participants became increasingly intertwined with the AV which created an identity and a way for others to know who they were. The AV was named an ‘evil twin’ (Mandy) which gave participants something to achieve and be proud of however also made them feel bad about themselves. Participants described a hostile side of the AV limiting their life and criticizing them. In some ways, the AV was described as developing into an abuser.

…the angel part of me […] wanted to be well, wanted to live a normal life, wanted to be 18 and out with friends and […] having fun. But that evil twin bit of me wouldn't allow that, or couldn't allow that to happen. (Mandy)

Participants spoke of the AV stripping away the external world, for example, missing large portions of the school. Whilst it is not within the scope of this paper, participants alluded to the fear of losing the AV, leaving them without a sense of self. They discussed finding it hard to think of life without the AV and feared returning to being internally unsafe. This appeared to act as a barrier to recovery.

## DISCUSSION

### Summary of the theory

The current study aimed to explore the link between adverse life experiences, emotions, the AV, and AN. The AV appeared to develop during periods of perceived relational and internal unsafety associated with adverse relational experiences (e.g., appearance‐related bullying). It emerged alongside AN symptoms and appeared to drive AN behaviours. Whilst the participants noted that the AV provided comfort, predictability, and friendship amid adverse experiences, the safety provided was conditional on adhering to strict behavioural rules. The AV reinforced obedience with praise and reassurance, aiding individuals in feeling successful and worthy. When individuals disobeyed the AV by breaking its rules, for example, around eating, the AV withdrew safety becoming increasingly hostile and belittling, revealing similar characteristics to abusers and bullies in childhood. This would activate individuals' unpleasant emotions from childhood, such as guilt and disgust. Following a similar strategy when attempting to placate abusers or bullies in childhood, individuals adopted a submissive position to the critical and attacking AV. Individuals would then often return to obeying the AV, thus maintaining the AN behaviours. Over time, the AV strengthened individuals' self‐identity as being ‘anorexic’. These findings mirror those of Forsén Mantilla et al. (2018), who reported sulking/submitting in response to a controlling and blaming eating disorder. Our findings are also consistent with the literature on auditory hallucinations (AHs) that highlights commonalities between the relationships people experience with their AHs and their social relationships (Birchwood et al., 2004). We contribute to this literature by proposing that early life traumas and relational dynamics are associated with characteristics of people's dynamics with the AV.

One key finding from the study was that traumatic experiences were often internalized by the participant which meant they heard the AV as if it was the abuser or bully. This may be linked to a process of dissociation where the individual disconnects emotionally and psychologically from a threatening experience (Pugh et al., 2018), leading dissociated content to be experienced as an internal voice (Longden et al., 2012).

A commonality between individuals was experiencing emotional abuse by caregivers and/or bullies. Research has suggested childhood emotional abuse has the greatest impact on ED symptomatology (Guillaume et al., 2016; Rai et al., 2019). Sexual and emotional abuse frequently co‐occur (Jonson‐Reid et al., 2003; Kim et al., 2017). Individuals had also experienced sexual abuse and described the AV as engaging in behaviours that were like grooming experiences. The present theory outlines a mechanism in which the relationship with the AV could perpetuate AN, that is, by mimicking previous abusive relational dynamics.

Individuals described an untrusting relationship with their own emotions. The AV was viewed positively in terms of its ability to protect the self by keeping individuals detached from feelings and disconnected from others. Wesselius et al. (2020) suggest children ‘self‐silence’ about negative childhood experiences. The present study's findings resonate with studies reporting individuals with EDs experience high levels of negative emotions but fail to express them (Fox & Froom, 2009; Henderson et al., 2019). Our findings also bear similarity to the proposal of Oldershaw et al. (2019) that a variety of factors such as early attachment experiences and relationships with peers lead individuals with AN to be overwhelmed by their own emotions. Oldershaw et al. argued that AN is a means of regulating emotional experience in a way that inadvertently interferes with the development of a coherent sense of self and an understanding of one's emotional needs.

Bullying victimization has been associated with a range of ED symptoms such as restricted eating, in both clinical and non‐clinical populations (Copeland et al., 2015; Day et al., (2021). This coheres with the experiences of individuals in the present study, all of whom had experienced bullying. Individuals reported that subsequent loneliness and not belonging created a need for companionship, which then arose in the form of the AV. Bullying in childhood is associated with an almost doubling of the likelihood of experiencing hearing voices (Løberg et al., 2019; Schreier et al., 2009). The AV could be seen as providing a friendship to meet unmet relatedness needs by promising socially accepted thinness (Banerjee & Dittmar, 2008).

Participants described the AV as giving them an acceptable identity, countering the belief that they were bad people. The AV might be seen initially as the individual's attempting to develop a false self that reduces the impact of trauma thus avoiding intolerable negative effect (McIntee & Crompton, 1997). However, as individuals engaged in recovery, the AV was seen as depriving them of opportunities to develop an identity away from the AN, for example, by encouraging individuals to restrict food intake. This is consistent with Williams et al.'s (2016) findings that participants described an intense fear that without AN, they would be no one. By defining themselves in terms of AN and equating their self‐worth with low BMI, individuals develop a reluctance to leave the AN creating a barrier to recovery (Lamoureux & Bottorff, 2005).

### Limitations and future research

This study explored the views of individuals with AN on whether they perceived interaction between the AV, negative childhood events, and emotions by asking them to retrospectively reflect on their experiences. Previous research has found that there may be associations between childhood trauma and AN (Molendijk et al., 2017). Exposure to this literature may have biased some of the reflections on life events made by participants. This may be further exacerbated by selection bias, where those participants who had subjectively experienced trauma may have been more likely to participate in this study. More traditional grounded theory has been criticized for assuming that researchers approach projects as a *tabula rasa* (Charmaz, 2014). Whilst these concepts guided theory across the research, a range of strategies were included to ensure that an a priori framework was not placed on the data.

All participants were recruited from social media; therefore, a lifetime history of AN was not confirmed by professional diagnosis. By considering interview and EDE‐Q data, participants were evaluated as to whether they had met the DSM‐5 criteria for AN in their lifetime. There was no cause to dispute the validity of participants' narratives, given the consistency of their stories and corroboration with research supervisors. Future research may want to recruit from NHS services to confirm the generalisability of findings to clinical settings.

The participants in this study all identified as female, and as such the results are limited in establishing whether similar processes arise for males with AN. There was also limited ethnic diversity in the sample, limiting our ability to draw out cultural aspects or experiences of discrimination that may be important. It would have been beneficial also to collect information about other aspects of diversity, including sexual and gender diversity, as well as neurodiversity; experiences of stigma, for example, can lead to internalized prejudices (e.g., Poštuvan et al., 2019), which could have some bearing on the nature of one's relationship with the AV. We are limited in our understanding of how the relationship between people with AN and AV might change after multiple decades as most participants in this study were in their twenties; there is evidence that many relevant factors, such as social networks, continue to deteriorate in those with longstanding AN (Robinson et al., 2015), and little is known about the AV in this population.

### Clinical implications

This study provides an account of the link between adverse life experiences, the AV, and AN. It corroborates existing theory and empirical findings in the field and provides additional insights: the AV appears to mirror the relational dynamics of early adverse experiences, such as abuse and bullying. The implications of this are that trauma‐focused and emotion‐focused interventions may be important when working with the AV. Clinicians should enquire about early traumatic events when working with individuals experiencing the AV in the context of AN, and more consideration should be given to the adaptation of therapeutic approaches to include addressing relational trauma. This might involve using therapeutic approaches that are relatively novel to this population such as grounding techniques for managing symptoms of trauma and experiential interventions aimed at supporting disrupted attachments associated with internal voices (e.g., empty‐chair confrontation of past abusers; Arntz, 2012; Pugh, 2019).

## CONCLUSION

As expressed by the theoretical model, the association between AV, emotions, and childhood trauma is complex. The current study proposes a theory grounded in interview data in which the AV provides a sense of safety amid life experiences that generate a sense of unsafety. However, the safety provided by the AV is conditional, and in this way mirrors a perfectionistic coping style. A further insight is that the relational dynamics of the AV appear to reflect the adversity encountered in earlier relational experiences. Findings reflect previous theoretical accounts of attachment, alongside factors associated with experiences of childhood trauma, AN, and the AV.

This paper has discussed the implications of these findings for clinicians working with individuals with AN, and for researching treatments novel to this area or adapting existing treatments to incorporate guidance for working with the AV in a way that considers the role of adverse life experiences.

## AUTHOR CONTRIBUTIONS

**Jet Morrison:** Conceptualization; data curation; formal analysis; investigation; methodology; project administration; writing – original draft; writing – review and editing. **Marc O. Williams:** Conceptualization; supervision; writing – review and editing. **John R. E. Fox:** Conceptualization; supervision; writing – review and editing.

## FUNDING INFORMATION

The authors declare that no funds, grants, or other support were received during the preparation of this manuscript.

## CONFLICT OF INTEREST

The authors have no relevant financial or non‐financial interests to disclose.

## Data Availability

Excerpts of interviews with participants are provided in the manuscript. Consent was not obtained for sharing full interview transcripts.

## References

[papt12416-bib-0001] American Psychiatric Association . (2013). Diagnostic and statistical manual of mental disorders (5th ed.). American Psychiatric Association.

[papt12416-bib-0003] Arntz, A. (2012). Imagery rescripting as a therapeutic technique: Review of clinical trials, basic studies, and research agenda. Journal of Experimental Psychopathology, 3(2), 189–208.

[papt12416-bib-0005] Banerjee, R. , & Dittmar, H. (2008). Individual differences in children's materialism: The role of peer relations. Personality and Social Psychology Bulletin, 34(1), 17–31. 17989212 10.1177/0146167207309196

[papt12416-bib-0006] Berg, K. C. , Peterson, C. B. , Frazier, P. , & Crow, S. J. (2012). Psychometric evaluation of the eating disorder examination and eating disorder examination‐questionnaire: A systematic review of the literature. International Journal of Eating Disorders, 45(3), 428–438. 21744375 10.1002/eat.20931 PMC3668855

[papt12416-bib-0007] Birchwood, M. A. X. , Gilbert, P. , Gilbert, J. , Trower, P. , Meaden, A. , Hay, J. , Murray, E. , & Miles, J. N. (2004). Interpersonal and role‐related schema influence the relationship with the dominant ‘voice'in schizophrenia: A comparison of three models. Psychological Medicine, 34(8), 1571–1580. 15724887 10.1017/s0033291704002636

[papt12416-bib-0016] Charmaz, K. C. (2006). Constructing grounded theory: A practical guide through qualitative analysis. Sage Publications Ltd.

[papt12416-bib-0017] Charmaz, K. (2014). Constructing grounded theory (2nd ed.). SAGE Publications Ltd.

[papt12416-bib-0019] Copeland, W. E. , Bulik, C. M. , Zucker, N. , Wolke, D. , Lereya, S. T. , & Costello, E. J. (2015). Does childhood bullying predict eating disorder symptoms? A prospective, longitudinal analysis. International Journal of Eating Disorders, 48(8), 1141–1149. 26337405 10.1002/eat.22459 PMC4715551

[papt12416-bib-0022] Creswell, J. W. , Hanson, W. E. , Clark Plano, V. L. , & Morales, A. (2007). Qualitative research designs: Selection and implementation. The Counseling Psychologist, 35(2), 236–264. 10.1177/0011000006287390

[papt12416-bib-0210] Day, S. , Bussey, K. , Trompeter, N. , & Mitchison, D. (2021). The impact of teasing and bullying victimization on disordered eating and body image disturbance among adolescents: A systematic review. Trauma, Violence, & Abuse, 23(3), 985–1006. 10.1177/1524838020985534 33461439

[papt12416-bib-0025] Dobrescu, S. R. , Dinkler, L. , Gillberg, C. , Rastam, M. , Gillberg, C. , & Wentz, E. (2019). Anorexia nervosa: 30‐year outcome. The British Journal of Psychiatry, 216, 1–8. 10.1192/bjp.2019.113 PMC7557598 31113504

[papt12416-bib-0029] Fairburn, C. G. , & Beglin, S. (2008). Eating disorder examination questionnaire (EDE‐Q 6.0). In C. G. Fairburn (Ed.), Cognitive behavior therapy and eating disorders (pp. 309–313). The Guilford Press.

[papt12416-bib-0032] Forsén Mantilla, E. , Clinton, D. , & Birgegård, A. (2018). Insidious: The relationship patients have with their eating disorders and its impact on symptoms, duration of illness, and self‐image. Psychology and Psychotherapy: Theory, Research and Practice, 91(3), 302–316. 10.1111/papt.12161 PMC6175392 29080248

[papt12416-bib-0033] Fox, J. R. E. , Federici, A. , & Power, M. J. (2012). Emotions and eating disorders. In J. R. E. Fox & K. P. Goss (Eds.), Eatingand its disorders (pp. 167–184). John Wiley and Sons, Ltd.

[papt12416-bib-0208] Fox, J. R. , & Froom, K. (2009). Eating disorders: A basic emotion perspective. Clinical Psychology & Psychotherapy: An International Journal of Theory & Practice, 16(4), 328–335. 10.1002/cpp.622 19639651

[papt12416-bib-0036] Glaser, B. , & Strauss, A. (1967). The discovery of grounded theory. Aldine.

[papt12416-bib-0038] Graham, M. R. , Tierney, S. , Chisholm, A. , & Fox, J. R. (2019). Perceptions of the “anorexic voice”: A qualitative study of health care professionals. Clinical Psychology & Psychotherapy, 26(6), 707–716. 10.1002/cpp.2393 31368595

[papt12416-bib-0207] Guillaume, S. , Jaussent, I. , Maïmoun, L. , Ryst, A. , Seneque, M. , Villain, L. , Hamroun, D. , Lefebvre, P. , Renard, E. , & Courtet, P. (2016). Associations between adverse childhood experiences and clinical characteristics of eating disorders. Scientific Reports, 6(1), 1–7. 27804994 10.1038/srep35761 PMC5090200

[papt12416-bib-0209] Henderson, Z. B. , Fox, J. R. , Trayner, P. , & Wittkowski, A. (2019). Emotional development in eating disorders: A qualitative metasynthesis. Clinical Psychology & Psychotherapy, 26(4), 440–457. 30889630 10.1002/cpp.2365 PMC6766861

[papt12416-bib-0041] Hibbs, R. , Pugh, M. , & Fox, J. R. (2020). Applying emotion‐focused therapy to work with the “anorexic voice” within anorexia nervosa: A brief intervention. Journal of Psychotherapy Integration, 31(4), 327–347.

[papt12416-bib-0042] Higbed, L. , & Fox, J. R. E. (2010). Illness perceptions in anorexia nervosa: A qualitative investigation. British Journal of Clinical Psychology, 49, 307–325. 10.1348/014466509X454598 19580703

[papt12416-bib-0204] Illing, V. , Tasca, G. A. , Balfour, L. , & Bissada, H. (2010). Attachment insecurity predicts eating disorder symptoms and treatment outcomes in a clinical sample of women. The Journal of Nervous and Mental Disease, 198(9), 653–659. 20823727 10.1097/NMD.0b013e3181ef34b2

[papt12416-bib-0046] Jonson‐Reid, M. , Drake, B. , Chung, S. , & Way, I. (2003). Cross‐type recidivism among child maltreatment victims and perpetrators. Child Abuse & Neglect, 27(8), 899–917. 10.1016/S0145-2134(03)00138-8 12951139

[papt12416-bib-0047] Kim, K. , Mennen, F. E. , & Trickett, P. K. (2017). Patterns and correlates of co‐occurrence among multiple types of child maltreatment. Child & Family Social Work, 22(1), 492–502. 29225485 10.1111/cfs.12268 PMC5720384

[papt12416-bib-0049] Kong, S. , & Bernstein, K. (2009). Childhood trauma as a predictor of eating psychopathology and its mediating variables in patients with eating disorders. Journal of Clinical Nursing, 18(13), 1897–1907. 10.1111/j.1365-2702.2008.02740.x 19638049

[papt12416-bib-0050] Lamoureux, M. M. , & Bottorff, J. L. (2005). “Becoming the real me”: Recovering from anorexia nervosa. Health Care for Women International, 26(2), 170–188. 10.1080/07399330590905602 15804915

[papt12416-bib-0052] Løberg, E. M. , Gjestad, R. , Posserud, M. B. , Kompus, K. , & Lundervold, A. J. (2019). Psychosocial characteristics differentiate non‐distressing and distressing voices in 10,346 adolescents. European Child & Adolescent Psychiatry, 28(10), 1353–1363. 10.1007/s00787-019-01292-x 30820670 PMC6785583

[papt12416-bib-0053] Longden, E. , Madill, A. , & Waterman, M. G. (2012). Dissociation, trauma, and the role of lived experience: Toward a new conceptualization of voice hearing. Psychological Bulletin, 138(1), 28–76. 10.1037/a0025995 22082488

[papt12416-bib-0054] Luce, K. H. , & Crowther, J. H. (1999). The reliability of the eating disorder examination—Self‐report questionnaire version (EDE‐Q). International Journal of Eating Disorders, 25(3), 349–351. 10192002 10.1002/(sici)1098-108x(199904)25:3<349::aid-eat15>3.0.co;2-m

[papt12416-bib-0056] McIntee, J. , & Crompton, I. (1997). The psychological effects of trauma on children. In Protecting children: Challenges and change (pp. 127–142). Arena Publishers. Aldershot, UK.

[papt12416-bib-0057] Messman‐Moore, T. L. , & Coates, A. A. (2007). The impact of childhood psychological abuse on adult interpersonal conflict: The role of early maladaptive schemas and patterns of interpersonal behavior. Journal of Emotional Abuse, 7(2), 75–92. 10.1300/J135v07n02_05

[papt12416-bib-0205] Mills, P. , Newman, E. F. , Cossar, J. , & Murray, G. (2015). Emotional maltreatment and disordered eating in adolescents: Testing the mediating role of emotion regulation. Child Abuse & Neglect, 39, 156–166. 25129874 10.1016/j.chiabu.2014.05.011

[papt12416-bib-0058] Molendijk, M. L. , Hoek, H. W. , Brewerton, T. D. , & Elzinga, B. M. (2017). Childhood maltreatment and eating disorder pathology: A systematic review and dose‐response meta‐analysis. Psychological Medicine, 47(8), 1402–1416. 10.1017/S0033291716003561 28100288

[papt12416-bib-0059] Mond, J. M. , Hay, P. J. , Rodgers, B. , Owen, C. , & Beumont, P. J. V. (2004). Validity of the Eating Disorder Examination Questionnaire (EDE‐Q) in screening for eating disorders in community samples. Behaviour Research and Therapy, 42(5), 551–567. 15033501 10.1016/S0005-7967(03)00161-X

[papt12416-bib-0062] National Institute for Health and Care Excellence . (2017). Eating disorders: Recognition and treatment. (NICE Clinical Guideline No. 69). Retrieved from: https://www.nice.org.uk/guidance/ng69/chapter/Recommendations#treating‐anorexia‐nervosa 28654225

[papt12416-bib-0064] Noordenbos, G. , Aliakbari, N. , & Campbell, R. (2014). The relationship among critical inner voices, low self‐esteem, and self‐criticism in eating disorders. Eating Disorders, 22(4), 337–351. 10.1080/10640266.2014.898983 24678635

[papt12416-bib-0066] Oldershaw, A. , Startup, H. , & Lavender, T. (2019). Anorexia nervosa and a lost emotional self: A psychological formulation of the development, maintenance, and treatment of anorexia nervosa. Frontiers in Psychology, 10, 219. 30886593 10.3389/fpsyg.2019.00219 PMC6410927

[papt12416-bib-0067] Pennebaker, J. W. , & Susman, J. R. (1988). Disclosure of traumas and psychosomatic processes. Social Science & Medicine, 26(3), 327–332. 3279521 10.1016/0277-9536(88)90397-8

[papt12416-bib-0069] Polivy, J. , & Herman, C. P. (2002). Causes of eating disorders. Annual Review of Psychology, 53(1), 187–213. 10.1146/annurev.psych.53.100901.135103 11752484

[papt12416-bib-0070] Poštuvan, V. , Podlogar, T. , Šedivy, N. Z. , & De Leo, D. (2019). Suicidal behaviour among sexual‐minority youth: A review of the role of acceptance and support. The Lancet Child & Adolescent Health, 3(3), 190–198. 30679139 10.1016/S2352-4642(18)30400-0

[papt12416-bib-0075] Pugh, M. (2016). The internal ‘anorexic voice’: A feature or fallacy of eating disorders? Advances in Eating Disorders: Theory, Research and Practice, 4, 75–83. 10.1080/21662630.2015.1116017

[papt12416-bib-0078] Pugh, M. (2019). Cognitive behavioural chairwork: Distinctive features (1st ed.). Routledge. 10.4324/9780429023927

[papt12416-bib-0077] Pugh, M. , & Waller, G. (2017). Understanding the ‘anorexic voice’ in anorexia nervosa. Clinical Psychology and Psychotherapy, 24, 670–676. 10.1002/cpp.2034 27435632

[papt12416-bib-0202] Pugh, M. , Waller, G. , & Esposito, M. (2018). Childhood trauma, dissociation, and the internal eating disorder ‘voice’. Child Abuse & Neglect, 86, 197–205. 30326368 10.1016/j.chiabu.2018.10.005

[papt12416-bib-0079] Racine, S. E. , & Wildes, J. E. (2015). Emotion dysregulation and anorexia nervosa: An exploration of the role of childhood abuse. International Journal of Eating Disorders, 48(1), 55–58. 25358997 10.1002/eat.22364 PMC4404145

[papt12416-bib-0080] Rabito‐Alcón, M. F. , Baile, J. I. , & Vanderlinden, J. (2021). Mediating factors between childhood traumatic experiences and eating disorders development: A systematic review. Children, 8(2), 114. 10.3390/children8020114 33561984 PMC7915652

[papt12416-bib-0083] Rai, T. , Mainali, P. , Raza, A. , Rashid, J. , & Rutkofsky, I. (2019). Exploring the link between emotional child abuse and anorexia nervosa: A psychopathological correlation. Cureus, 11(8). 10.7759/cureus.5318 PMC6777933 31598427

[papt12416-bib-0084] Robinson, P. H. , Kukucska, R. , Guidetti, G. , & Leavey, G. (2015). Severe and enduring anorexia nervosa (SEED‐AN): A qualitative study of patients with 20+ years of anorexia nervosa. European Eating Disorders Review, 23(4), 318–326. 26059633 10.1002/erv.2367

[papt12416-bib-0087] Scheller‐Gilkey, G. , Moynes, K. , Cooper, I. , Kant, C. , & Miller, A. H. (2004). Early life stress and PTSD symptoms in patients with comorbid schizophrenia and substance abuse. Schizophrenia Research, 69(2–3), 167–174. 15469190 10.1016/s0920-9964(03)00188-9

[papt12416-bib-0091] Schreier, A. , Wolke, D. , Thomas, K. , Horwood, J. , Hollis, C. , Gunnell, D. , Lewis, G. , Thompson, A. , Zammit, S. , Duffy, L. , Salvi, G. , & Harrison, G. (2009). Prospective study of peer victimization in childhood and psychotic symptoms in a nonclinical population at age 12 years. Archives of General Psychiatry, 66(5), 527–536. 10.1001/archgenpsychiatry.2009.23 19414712

[papt12416-bib-0094] Smyth, J. M. , Heron, K. E. , Wonderlich, S. A. , Crosby, R. D. , & Thompson, K. M. (2008). The influence of reported trauma and adverse events on eating disturbance in young adults. International Journal of Eating Disorders, 41(3), 195–202. 10.1002/eat.20490 18008320

[papt12416-bib-0206] Tasca, G. A. , Szadkowski, L. , Illing, V. , Trinneer, A. , Grenon, R. , Demidenko, N. , Krysanski, V. , Balfour, L. , & Bissada, H. (2009). Adult attachment, depression, and eating disorder symptoms: The mediating role of affect regulation strategies. Personality and Individual Differences, 47(6), 662–667.

[papt12416-bib-0100] Thakkar, R. R. , & McCanne, T. R. (2000). The effects of daily stressors on physical health in women with and without a childhood history of sexual abuse. Child Abuse & Neglect, 24(2), 209–221. 10695516 10.1016/s0145-2134(99)00129-5

[papt12416-bib-0101] Tierney, S. , & Fox, J. R. (2010). Living with the “anorexic voice”: A thematic analysis. Psychology and Psychotherapy: Theory, Research and Practice, 83, 243–254. 10.1348/147608309X480172 20109280

[papt12416-bib-0102] Tierney, S. , & Fox, J. R. (2011). Trapped in a toxic relationship: Comparing the views of women living with anorexia nervosa to those experiencing domestic violence. Journal of Gender Studies, 20(1), 31–41. 10.1080/09589236.2011.542018

[papt12416-bib-0111] Villarroel, A. M. , Penelo, E. , Portell, M. , & Raich, R. M. (2012). Childhood sexual and physical abuse in Spanish female undergraduates: Does it affect eating disturbances? European Eating Disorders Review, 20(1), e32–e41. 10.1002/erv.1086 21394836

[papt12416-bib-0113] Wesselius, H. , Bosch, I. , van Hastenberg, L. , Simons, J. , Kuiper, C. , & van der Helm, P. (2020). A new perspective on extreme recurring anorexia and its treatment: A preliminary study. Journal of Prenatal & Perinatal Psychology & Health, 34(5), 369–393.

[papt12416-bib-0115] Williams, K. , King, J. , & Fox, J. R. E. (2016). Sense of self and anorexia nervosa: A grounded theory. Psychology and Psychotherapy: Theory, Research and Practice, 89(2), 211–228. 10.1111/papt.12068 26179295

[papt12416-bib-0203] Williams, S. , & Reid, M. (2010). Understanding the experience of ambivalence in anorexia nervosa: The maintainer's perspective. Psychology and Health, 25(5), 551–567. 20204933 10.1080/08870440802617629

[papt12416-bib-0114] Williams, S. , & Reid, M. (2012). ‘It's like there are two people in my head’: A phenomenological exploration of anorexia nervosa and its relationship to the self. Psychology and Health, 27, 798–815. 10.1080/08870446.2011.595488 21736500

[papt12416-bib-0116] Willig, C. (2013). Grounded theory methodology. In Introducing qualitative research in psychology (p. 3). Open University Press, Berkshire.

[papt12416-bib-0120] Zipfel, S. , Wild, B. , Groß, G. , Friedrich, H.‐C. , Teufel, M. , Schellberg, D. , Giel, K. E. , Zwaan, M. D. , Dinkel, A. , Herpertz, S. , Burgmer, M. , Löwe, B. , Tagay, S. , Wietersheim, J. V. , Zeeck, A. , Schade‐Brittinger, C. , Schauenburg, H. , & Herzog, W. (2014). Focal psychodynamic therapy, cognitive behaviour therapy, and optimised treatment as usual in outpatients with anorexia nervosa (ANTOP study): Randomised controlled trial. The Lancet, 383(9912), 127–137. 10.1016/S0140-6736(13)61746-8 24131861

